# Cerebral microbleeds on susceptibility-weighted imaging, cognitive domains, and serum neurofilament light chain in adults with hypertension

**DOI:** 10.3389/fneur.2026.1909512

**Published:** 2026-09-03

**Authors:** Ziyin Li, Xiaobin Zhao, Dandan Zhou, Cui Zhao

**Affiliations:** 1 Department of General Practice, The Affiliated Hospital of Chengde Medical College, Chengde, Hebei, China; 2 Department of Radiology, The Affiliated Hospital of Chengde Medical College, Chengde, Hebei, China

**Keywords:** cerebral microbleeds, cognitive domains, hypertension, mild cognitive impairment, neurofilament light chain, susceptibility-weighted imaging

## Abstract

**Background:**

Hypertension is associated with cognitive impairment, and cerebral microbleeds (CMBs) may reflect hemorrhagic small-vessel injury. We examined whether CMB burden and location were associated with global and domain-specific cognition in adults with primary hypertension and whether serum neurofilament light chain (sNFL) provided additional discrimination for identifying mild cognitive impairment (MCI) beyond CMB count.

**Methods:**

In this single-center cross-sectional study, 173 hospitalized adults aged 31–95 years with a pre-existing diagnosis of primary hypertension underwent brain magnetic resonance imaging (MRI) with susceptibility-weighted imaging, the Montreal Cognitive Assessment (MoCA), and sNFL measurement by enzyme-linked immunosorbent assay (ELISA). Six MoCA-derived cognitive domain z-scores were calculated. CMB burden was categorized as 0, 1–4, or ≥5 lesions. Multivariable logistic and linear regression models were adjusted for age, sex, education, systolic blood pressure, diabetes, and prior stroke. Sensitivity analyses additionally adjusted for other cerebral small vessel disease markers, modeled the CMB count as a continuous variable, and excluded participants with prior stroke.

**Results:**

CMBs were detected in 82 patients (47.4%) and MCI in 83 patients (48.0%). Greater CMB burden was independently associated with MCI (adjusted odds ratio (OR): 4.53, 95% CI 1.95–10.55). After covariate adjustment and false-discovery-rate correction, greater CMB burden was associated with lower executive function, orientation, attention, and visuospatial function scores, but not with memory or language. Cognitive domain scores did not differ according to CMB location. CMB count and sNFL were inversely correlated with cognitive performance. The areas under the curve for identifying MCI were 0.683 for CMB count, 0.794 for sNFL, and 0.792 for the combined model. The combined model had a larger area under the curve than CMB count alone (*p* < 0.001) but did not differ from sNFL alone (*p* = 0.768).

**Conclusion:**

In adults with primary hypertension, greater CMB burden was associated with MCI and selected cognitive domains, whereas cognitive performance did not differ according to CMB location. sNFL had a numerically higher area under the curve than CMB count, and adding CMB count did not improve discrimination beyond sNFL. These findings may help characterize cognitive vulnerability in hypertension; therefore, prospective validation is required before clinical application.

## Introduction

Hypertension ranks among the most common chronic conditions in older adults and is a leading modifiable contributor to cognitive impairment (1). In Chinese patients with hypertension, the pooled prevalence of cognitive impairment is approximately 37.6%, and the likelihood of impairment increases across hypertension stages (2). A large part of this burden occurs at the stage of mild cognitive impairment (MCI), an early and potentially reversible window before overt dementia during which vascular risk factor management may still be beneficial (2, 3). Among community-dwelling hypertensive older adults, the reported prevalence of MCI approaches 35% (4). Hypertension may affect cognition partly through cerebral small vessel disease, involving arteriolar remodeling, endothelial dysfunction, impaired cerebral autoregulation, and blood–brain barrier injury (1). As the vascular risk that drives this trajectory can be treated, recognizing affected individuals early offers a practical chance to slow further decline (4).

Cerebral microbleeds (CMBs) are one of the established imaging markers of cerebral small vessel disease. On susceptibility-weighted imaging (SWI), they appear as small, round areas of signal loss produced by the paramagnetic effect of blood-degradation products, making SWI the preferred sequence for detecting and localizing these lesions (5). The anatomical distribution of CMBs also carries etiological meaning. Deep and infratentorial lesions point toward hypertensive arteriopathy, whereas strictly lobar lesions more often reflect cerebral amyloid angiopathy (6).

The clinical importance of CMBs lies in their relationship with cognition. Their presence is associated with cognitive decline and incident dementia, and the effect may strengthen as the lesion count rises (7). Evidence further links CMBs to a higher risk of Alzheimer disease and cognitive impairment (8). Hypertensive arteriolar injury and CMBs may also mark broader microvascular damage that disrupts the brain networks supporting cognition (1). In hypertensive patients without prior stroke, a heavier microbleed burden is independently related to MCI and poorer cognitive performance (9). However, it remains unclear whether cognition is more closely associated with total CMB burden or anatomical distribution, particularly in hypertension-specific populations and across individual cognitive domains. Clarifying these relationships in a hypertensive population could help direct cognitive screening toward those most likely to benefit.

Serum neurofilament light chain (sNFL) is a blood marker of neuroaxonal injury. Higher sNFL concentrations have been associated with poorer global and domain-specific cognition in older adults and individuals with vascular MCI (10–12). Population-based data have also linked higher blood NfL to greater overall cerebral small vessel disease (CSVD) burden and incident dementia, although its associations with individual magnetic resonance imaging (MRI) markers are not uniform (13, 14). CMBs capture focal hemorrhagic small-vessel injury, whereas sNFL may reflect downstream or more diffuse axonal damage; evaluating both markers may therefore help determine whether they provide complementary information on cognitive impairment. By reflecting neuronal injury, sNFL may complement imaging by capturing biological alterations associated with cognitive impairment.

This study examined, in adults with hypertension, the relationship between SWI-detected CMB burden and location and performance across MoCA-derived cognitive domains, and assessed whether sNFL could improve discrimination of MCI status. This study hypothesized that greater CMB burden would be associated with MCI and lower cognitive domain scores and that sNFL would provide complementary discriminatory information beyond CMB count; associations with CMB location were also examined.

## Materials and methods

### Study design and participants

This single-center, cross-sectional study consecutively screened patients with primary hypertension who were admitted to the Department of General Practice of The Affiliated Hospital of Chengde Medical College between December 2021 and November 2022. Eligible patients had a pre-existing diagnosis of primary hypertension, underwent brain MRI that included an SWI sequence, and completed cognitive assessment. Patients were excluded if they had secondary hypertension, an established non-vascular cause of dementia such as Alzheimer disease, another primary neurological or psychiatric disorder that prevented cognitive testing, active malignancy, severe hepatic, renal, or cardiac insufficiency, or a contraindication to MRI. A total of 173 patients were included. The study protocol was reviewed and approved by the Ethics Committee of The Affiliated Hospital of Chengde Medical College (approval number: CYFYLL2023410) and was performed in accordance with the principles of the Declaration of Helsinki. Written informed consent was obtained from all participants before enrollment.

### Clinical data and blood pressure assessment

Demographic and clinical data were recorded, including age, sex, years of education, diabetes, and prior stroke. All participants had a pre-existing diagnosis of primary hypertension before enrollment. Office blood pressure was measured at enrollment after the patient had rested in a seated position, and systolic and diastolic values were recorded. Hypertension severity was graded as grades 1–3 according to the Chinese guidelines for the prevention and treatment of hypertension (15).

### Brain MRI and cerebral microbleed assessment

Brain MRI was performed using a Siemens Verio 3.0-T MRI scanner (Siemens, Germany). The SWI sequence was acquired with a repetition time of 27 ms, an echo time of 20 ms, a flip angle of 15°, a slice thickness of 2 mm, a field of view of 220 × 220 mm^2^, and an acquisition matrix of 320 × 240.

CMBs were defined as small, round or ovoid foci of signal loss on SWI, generally 2–5 mm in diameter, distinct from vascular flow voids, calcification, and symmetrical basal ganglia mineralization, following consensus criteria (16). Each lesion was mapped using the Microbleed Anatomical Rating Scale (17). The total CMB count was recorded, and lesions were classified by location, with each patient assigned to one of five categories—no CMB, lobar, deep, infratentorial, or mixed. CMB burden was stratified into three categories: 0, 1 to 4, and 5 or more lesions (9, 18). White matter hyperintensities were graded with the Fazekas scale (19). Lacunes and enlarged perivascular spaces were identified using standard neuroimaging definitions (20). Images were read independently by two experienced readers who were blinded to the clinical data, and inter-reader agreement for CMB detection and location classification was quantified separately using the kappa statistic.

### Cognitive assessment and group definition

Global cognition and cognitive domains were assessed with the Montreal Cognitive Assessment (MoCA) (21). One point was added to the MoCA total for participants with 12 years of education or fewer. Six MoCA-derived domain scores, including memory, executive function, attention, language, visuospatial function, and orientation, were calculated according to the published MoCA Index Score framework. The memory index score comprised delayed free recall, category-cued recall, and multiple-choice recognition, weighted as 3, 2, and 1 points per word, respectively, with a possible score of 0–15. The executive function index score comprised the modified trail-making task, clock drawing, forward and backward digit span, letter-A tapping, serial-7 subtraction, letter fluency, and abstraction, with a possible score of 0–13. The attention index score comprised forward and backward digit span, letter-A tapping, serial-7 subtraction, sentence repetition, and words recalled during the two immediate-recall trials, with a possible score of 0–18. The language index score comprised naming, sentence repetition, and letter fluency, with a possible score of 0–6. The visuospatial function index score comprised cube copying, clock drawing, and naming, with a possible score of 0–7. The orientation index score was the sum of the date, month, year, day, place, and city items, with a possible score of 0–6 (22–24). Each raw domain score was converted to a study-sample standardized z-score using the formula *z* = (individual raw domain score—study-sample mean)/study-sample standard deviation, with higher z-scores indicating better cognitive performance. The education adjustment was applied only to the MoCA total score and not to the individual domain scores. The construct validity of these MoCA-derived index scores has previously been evaluated against corresponding domains from comprehensive neuropsychological batteries (23, 24).

MCI was defined as an education-adjusted MoCA total score below 26 in participants with preserved activities of daily living and without a clinical diagnosis of dementia, whereas a total score of 26 or higher defined normal cognition (21, 25).

### sNFL measurement

Fasting venous blood was collected, centrifuged within 2 h, and stored at −80 °C until analysis. Serum NfL was measured using a high-sensitivity commercial enzyme-linked immunosorbent assay (CUSABIO Biotech, Wuhan, China). Samples were diluted before measurement according to the manufacturer’s protocol, and results were expressed in pg./mL.

### Statistical analysis

Continuous variables were summarized as means with standard deviations or medians with interquartile ranges according to their distribution and were compared using the *t*-test or a one-way analysis of variance for normally distributed data and the Mann–Whitney U test or Kruskal–Wallis test otherwise. Categorical variables were summarized as counts with percentages and compared using the chi-square test or the Fisher–Freeman–Halton exact test, as appropriate. Effect sizes for Kruskal–Wallis comparisons were expressed as epsilon-squared, and those for categorical comparisons were expressed as Cramér’s V. Cognitive domain z-scores were compared across CMB burden strata and CMB location categories. Trends across increasing CMB burden strata were evaluated using the Jonckheere–Terpstra test, with the effect size calculated as *r* = Z/√N. Separate multivariable linear regression models were fitted for each cognitive domain z-score, with CMB burden entered as an ordinal variable and adjusted for age, sex, education, systolic blood pressure, diabetes, and prior stroke. Adjusted regression coefficients and 95% confidence intervals were reported. For each family of analyses involving the six cognitive domains, *p*-values were adjusted using the Benjamini–Hochberg false discovery rate procedure, and both raw and false discovery rate (FDR)-adjusted *p-*values were reported.

Spearman correlation analysis was used to examine the associations between CMB count and sNFL as well as cognitive domain z-scores and the MoCA total score; approximate 95% confidence intervals for the correlation coefficients were obtained using Fisher’s z transformation. CMB burden was entered as an ordinal variable in the univariable and multivariable logistic regression models estimating its association with MCI. The multivariable model was adjusted for age, sex, education, systolic blood pressure, diabetes, and prior stroke. Before fitting the primary multivariable model, multicollinearity was assessed using tolerance and the variance inflation factor. Linearity of the logit for age, education, and systolic blood pressure was assessed using Box–Tidwell interaction terms; because education included zero values, 1 was added before logarithmic transformation. Model fit was evaluated using the Hosmer–Lemeshow test, −2 log likelihood, Cox–Snell *R*^2^, and Nagelkerke *R*^2^. Standardized residuals, leverage values, and Cook’s distances were examined, and a sensitivity analysis was performed after excluding observations with an absolute standardized residual greater than 3.

Additional sensitivity analyses adjusted for Fazekas grade, lacune count, and perivascular space grade entered CMB count as a continuous variable and restricted the analysis to participants without prior stroke. The receiver operating characteristic curves were used to evaluate CMB count, sNFL, and their combination for the identification of MCI, and areas under the curve were compared using the DeLong test. Missing data were not imputed; descriptive analyses used the available data for each variable, and multivariable analyses were based on complete cases. All tests were two-sided, and a *p-*value of < 0.05 was considered statistically significant. In the main text, *p*-values below 0.001 were reported as *p* < 0.001. Statistical analyses were performed using SPSS version 26.0 and R.

## Results

### Study population and baseline characteristics

**Figure 1:**
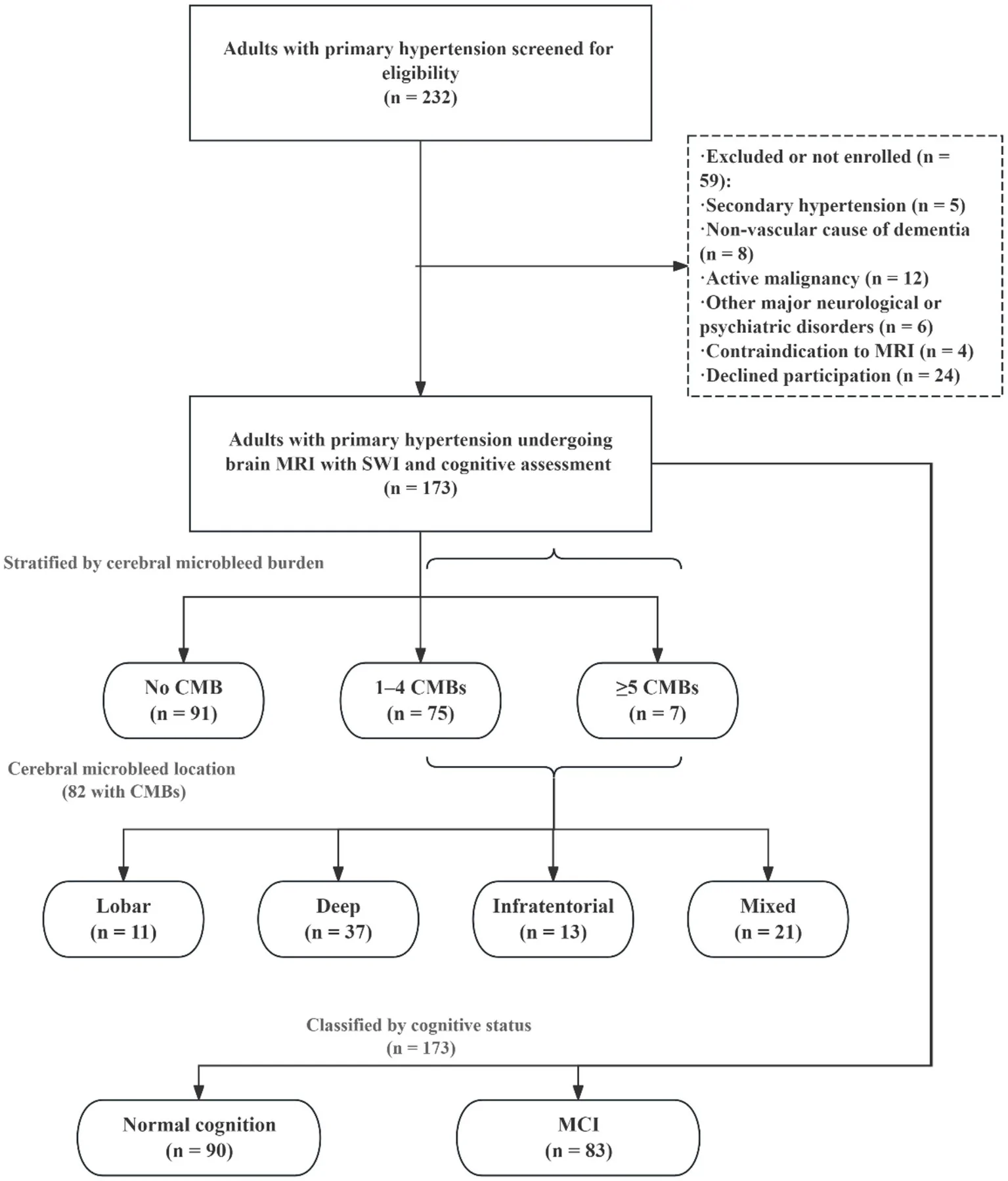
Participant screening, inclusion, and grouping. A total of 232 patients were screened, and 173 were included in the analysis. Grouping by CMB burden (0, 1–4, ≥5) and by cognitive status (normal cognition, MCI). CMB, cerebral microbleed; MCI, mild cognitive impairment.

A total of 232 patients with primary hypertension were screened. Of these, 59 were not included: 5 had secondary hypertension, 8 had an established non-vascular cause of dementia, 12 had active malignancy, 6 had another major neurological or psychiatric disorder, 4 had a contraindication to MRI, and 24 declined participation. The remaining 173 patients were included in the analysis (Figure 1). Sex was missing for one participant and hypertension grade for 12 participants; all other variables included in the primary analyses were complete.

On stratification by CMB burden, 91 patients had no CMB, 75 had 1–4 CMBs, and 7 had ≥5 CMBs; 83 patients (48.0%) were classified as having MCI. Across the three burden strata, median age increased from 67 to 70 and 78 years (ε^2^ = 0.053; *p* = 0.004), median systolic blood pressure from 132.00 to 140.00 and 171.00 mmHg (ε^2^ = 0.051; *p* = 0.005), median Fazekas grade from 1.00 to 1.00 and 3.00 (ε^2^ = 0.208; *p* < 0.001), and median sNFL from 20.10 to 41.00 and 59.00 pg./mL (ε^2^ = 0.287; *p* < 0.001), whereas median MoCA total score decreased from 27.00 to 25.00 and 25.00 (ε^2^ = 0.152; *p* < 0.001). The proportions of diabetes (25.27, 52.00, and 0.00%; Cramér’s V = 0.312; *p* < 0.001) and prior stroke (48.35, 62.67, and 100.00%; Cramér’s V = 0.228; *p* = 0.008), as well as the median perivascular space grade (ε^2^ = 0.075; *p* < 0.001), also differed across the burden strata. Results for the remaining characteristics are presented in Table 1.

**Table 1:** Baseline characteristics stratified by CMB burden.

Characteristic	Overall (*n* = 173)	No CMB (*n* = 91)	1–4 CMBs (*n* = 75)	≥5 CMBs (*n* = 7)	Effect size	*P*
Age (years)	69.00 (64.00, 78.00)	67.00 (61.50, 74.00)	70.00 (66.00, 80.00)	78.00 (64.00, 87.00)	ε^2^ = 0.053	0.004
Male sex, *n* (%)	89 (51.74)	44 (48.89)	41 (54.67)	4 (57.14)	V = 0.061	0.741
Education (years)	9.00 (6.00, 9.00)	9.00 (6.00, 9.00)	9.00 (6.00, 12.00)	6.00 (6.00, 9.00)	ε^2^ = 0.000	0.399
SBP (mmHg)	138.00 (127.00, 150.00)	132.00 (126.00, 146.50)	140.00 (130.00, 150.00)	171.00 (139.50, 179.00)	ε^2^ = 0.051	0.005
DBP (mmHg)	78.00 (72.00, 86.00)	75.00 (71.50, 87.00)	80.00 (72.00, 84.50)	90.00 (75.00, 103.50)	ε^2^ = 0.004	0.253
Hypertension grade, *n* (%)					V = 0.046	0.811
Grade 1	3 (1.86)	2 (2.44)	1 (1.39)	0 (0.00)		
Grade 2	15 (9.32)	8 (9.76)	6 (8.33)	1 (14.29)		
Grade 3	143 (88.82)	72 (87.80)	65 (90.28)	6 (85.71)		
Diabetes, *n* (%)	62 (35.84)	23 (25.27)	39 (52.00)	0 (0.00)	V = 0.312	<0.001
Prior stroke, *n* (%)	98 (56.65)	44 (48.35)	47 (62.67)	7 (100.00)	V = 0.228	0.008
Fazekas grade	1.00 (0.00, 2.00)	1.00 (0.00, 1.00)	1.00 (1.00, 2.00)	3.00 (3.00, 3.00)	ε^2^ = 0.208	<0.001
Lacune count	0.00 (0.00, 1.00)	0.00 (0.00, 1.00)	0.00 (0.00, 1.00)	1.00 (0.00, 2.00)	ε^2^ = 0.013	0.126
PVS grade	2.00 (1.00, 3.00)	2.00 (1.00, 2.00)	2.00 (1.00, 3.00)	3.00 (3.00, 3.50)	ε^2^ = 0.075	<0.001
MoCA (points)	26.00 (24.00, 27.00)	27.00 (25.00, 28.00)	25.00 (23.50, 26.00)	25.00 (24.50, 26.00)	ε^2^ = 0.152	<0.001
sNFL (pg/mL)	29.00 (17.90, 46.10)	20.10 (14.70, 30.60)	41.00 (28.15, 70.55)	59.00 (33.60, 109.25)	ε^2^ = 0.287	<0.001

Continuous variables are presented as median (IQR), and categorical variables are presented as *n* (%). The Kruskal–Wallis test was used for continuous variables; Fisher–Freeman–Halton exact tests were used for categorical variables.

Effect sizes are reported as epsilon-squared (ε^2^) for the Kruskal–Wallis tests and Cramér’s V for categorical comparisons. Sex data were available for 172 participants (90, 75, and 7 across the CMB burden groups), and hypertension grade was available for 161 participants (82, 72, and 7, respectively).

CMB, cerebral microbleed; SBP, systolic blood pressure; DBP, diastolic blood pressure; PVS, perivascular spaces; MoCA, Montreal Cognitive Assessment; sNFL, serum neurofilament light chain; SD, standard deviation; IQR, interquartile range.

### Detection and distribution of CMBs

**Figure 2:**
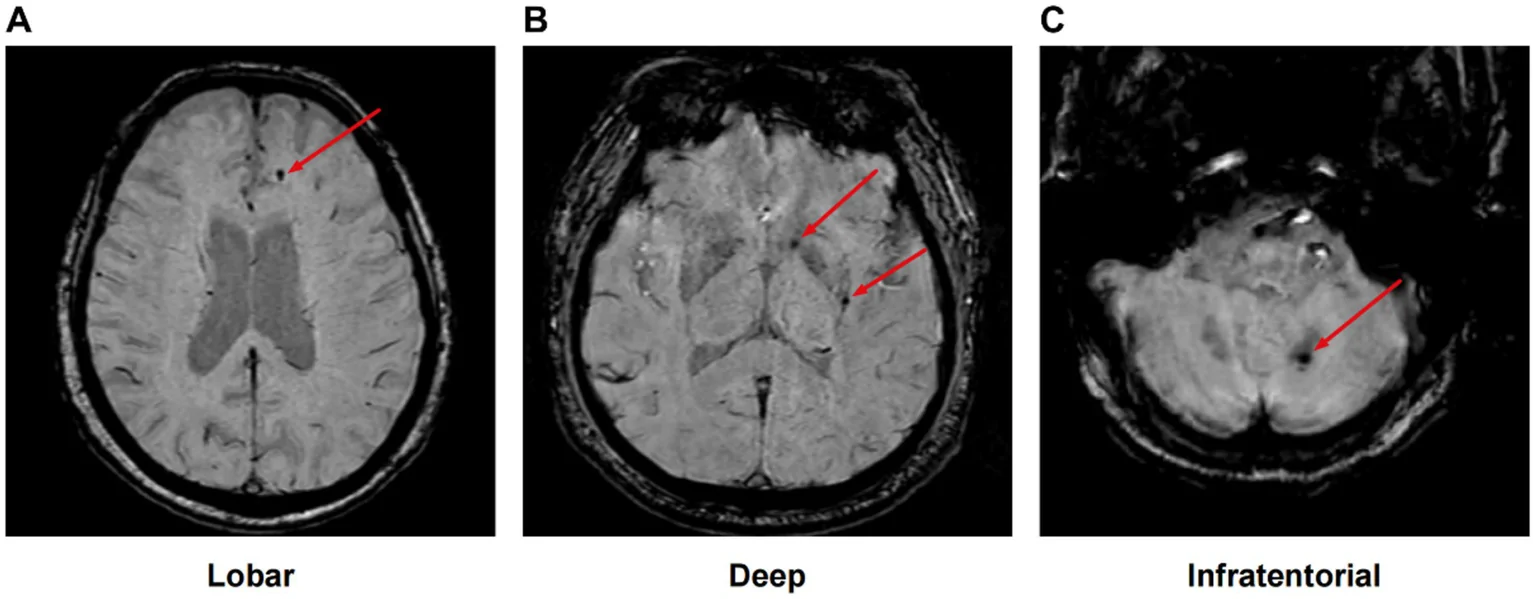
Representative CMBs on susceptibility-weighted imaging. Panels **(A–C)** show representative cerebral microbleeds (CMBs) in lobar, deep, and infratentorial locations, respectively. Arrows indicate the location of representative microbleeds.

CMBs were detected in 82 of the 173 patients (47.4%), and the CMB count ranged from 0 to 7. Among the 82 participants with CMBs, 11 (13.4%) had lobar CMBs, 37 (45.1%) had deep CMBs, 13 (15.9%) had infratentorial CMBs, and 21 (25.6%) had mixed-location CMBs. Representative SWI images of lobar, deep, and infratentorial CMBs are shown in Figure 2. The mixed-location category is not illustrated because demonstrating CMBs in more than one anatomical region would require multiple axial sections rather than a single representative image. Inter-reader agreement was 0.72 for CMB detection and 0.74 for CMB location classification.

### CMBs and cognitive domains

**Figure 3:**
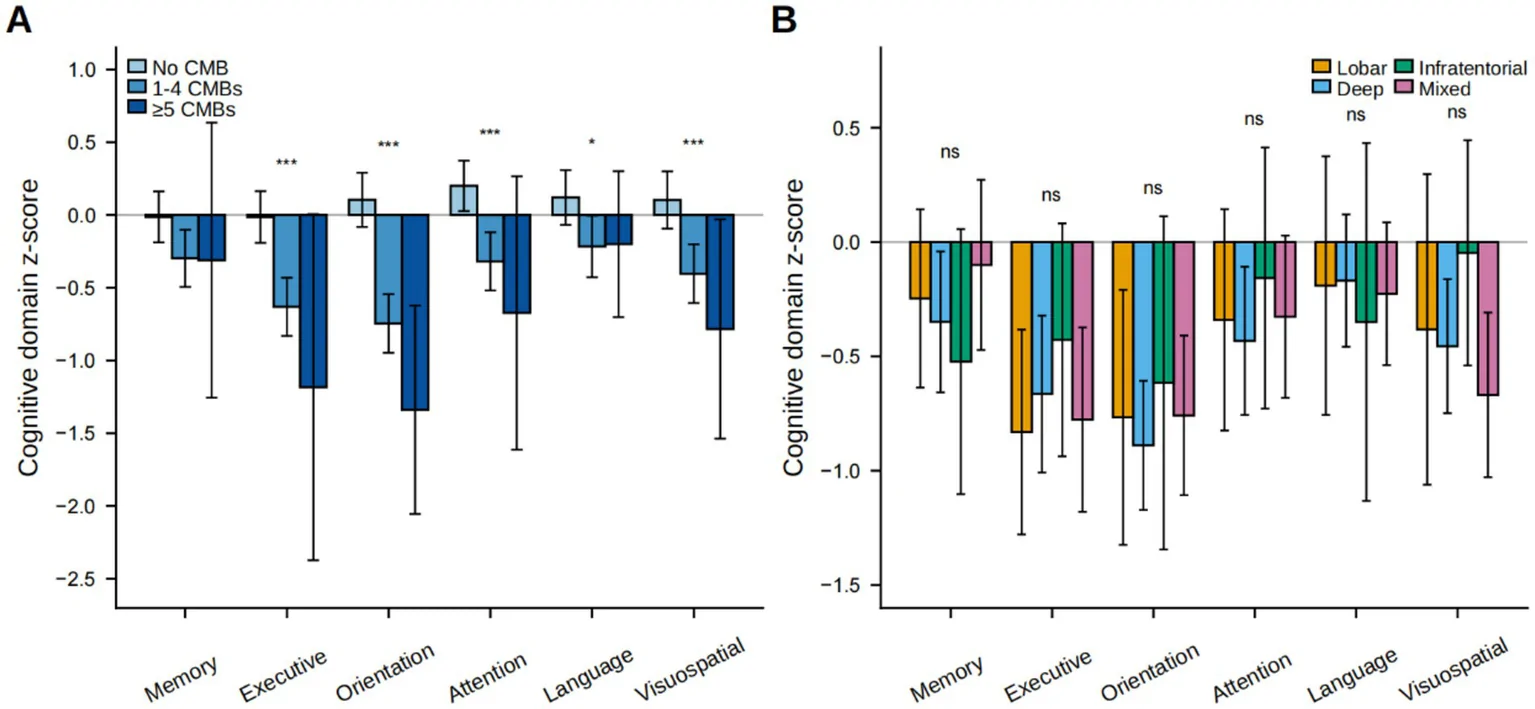
Cognitive domain *z*-scores. **(A)** CMB burden (0 CMB, *n* = 91; 1–4 CMBs, *n* = 75; ≥5 CMBs, *n* = 7); **(B)** CMB location (lobar, *n* = 11; deep, *n* = 37; infratentorial, *n* = 13; mixed, *n* = 21). Significance was assessed using the Jonckheere–Terpstra trend test for CMB burden and the Kruskal–Wallis test for CMB location. *p* values were adjusted using the Benjamini–Hochberg false discovery rate procedure for analyses involving multiple cognitive domains. *p < 0.05, **p < 0.01, ***p < 0.001; ns, not significant. CMB, cerebral microbleed; CI, confidence interval.

Cognitive domain z-scores decreased with increasing CMB burden (Figure 3A). After FDR correction, the decreasing trends remained significant for memory (*r* = −0.169; raw *p* = 0.026; FDR-adjusted *p* = 0.026), executive function (*r* = −0.357; raw *p* < 0.001; FDR-adjusted *p* < 0.001), orientation (*r* = −0.464; raw *p* < 0.001; FDR-adjusted *p* < 0.001), attention (*r* = −0.312; raw *p* < 0.001; FDR-adjusted *p* < 0.001), language (*r* = −0.170; raw *p* = 0.025; FDR-adjusted *p* = 0.026), and visuospatial function (*r* = −0.282; raw *p* < 0.001; FDR-adjusted *p* < 0.001) (Supplementary Table S1).

In the adjusted linear regression models, each one-category increase in CMB burden was associated with lower executive function (adjusted *β* = −0.463, 95% CI − 0.657 to −0.270; FDR-adjusted *p* < 0.001), orientation (adjusted *β* = −0.543, 95% CI − 0.740 to −0.346; FDR-adjusted *p* < 0.001), attention (adjusted *β* = −0.383, 95% CI − 0.587 to −0.178; FDR-adjusted *p* < 0.001), and visuospatial function scores (adjusted *β* = −0.248, 95% CI − 0.460 to −0.036; FDR-adjusted *p* = 0.033). The adjusted associations with memory (adjusted *β* = −0.050, 95% CI − 0.254 to 0.155; FDR-adjusted *p* = 0.632) and language (adjusted *β* = −0.117, 95% CI − 0.329 to 0.095; FDR-adjusted *p* = 0.334) were not statistically significant (Supplementary Table S2).

Among participants with CMBs, none of the six cognitive domains differed across the lobar, deep, infratentorial, and mixed-location groups. The raw *p*-values ranged from 0.261 to 0.969, all FDR-adjusted *p*-values were 0.969, and epsilon-squared values ranged from 0.000 to 0.013 (Figure 3B and Supplementary Table S1).

### Correlation between cerebral microbleed count and sNFL and cognition

**Figure 4:**
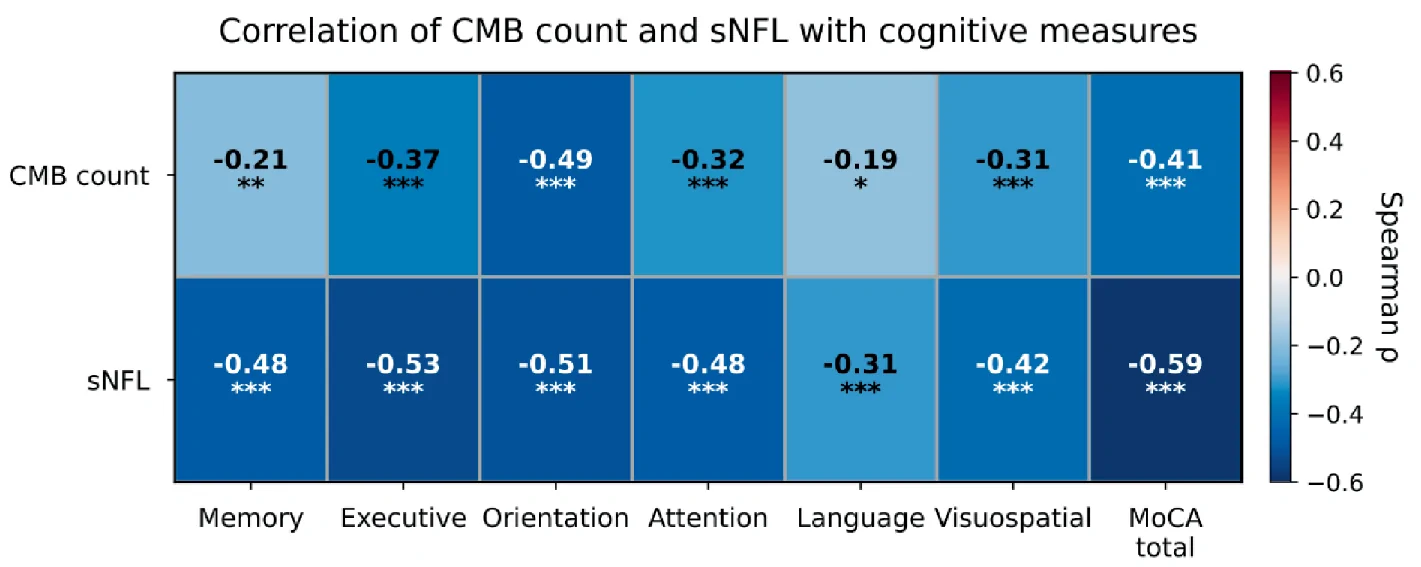
Correlations of CMB count and sNFL with cognitive measures. Cell values, Spearman *ρ*. Analyses included 173 participants. *p* values were adjusted using the Benjamini–Hochberg false discovery rate procedure for analyses involving multiple cognitive domains. *p < 0.05, **p < 0.01, ***p < 0.001. CMB, cerebral microbleed; sNFL, serum neurofilament light chain; MoCA, Montreal Cognitive Assessment.

CMB count and sNFL were each inversely correlated with all six cognitive domain z-scores and with the MoCA total score (Figure 4 and Supplementary Table S1). For the six cognitive domains, the FDR-adjusted *p*-values ranged from <0.001 to 0.010 for CMB count and were all <0.001 for sNFL. The strongest domain-level correlation with CMB count was observed for orientation (*ρ* = −0.493, 95% CI − 0.598 to −0.371; raw *p* < 0.001; FDR-adjusted *p* < 0.001), whereas its correlation with the MoCA total score was *ρ* = −0.414 (95% CI − 0.531 to −0.283; *p* < 0.001). For sNFL, the strongest correlation was with the MoCA total score (*ρ* = −0.587, 95% CI − 0.677 to −0.479; *p* < 0.001), followed by executive function (*ρ* = −0.534, 95% CI − 0.633 to −0.418; raw *p* < 0.001; FDR-adjusted *p* < 0.001).

### Independent factors associated with MCI

In the multivariable logistic regression analysis, higher CMB burden (adjusted OR 4.53, 95% CI 1.95–10.55; *p* < 0.001) and older age (adjusted OR 1.16 per year, 95% CI 1.10–1.23; *p* < 0.001) were associated with higher odds of MCI, whereas more years of education were associated with lower odds (adjusted OR 0.81 per year, 95% CI 0.71–0.91; p < 0.001). Sex (*p* = 0.604), systolic blood pressure (*p* = 0.562), diabetes (*p* = 0.559), and prior stroke (*p* = 0.118) were not independently associated with MCI (Table 2). The association between CMB burden and MCI remained statistically significant after additional adjustment for Fazekas grade, lacune count, and perivascular space grade (adjusted OR: 4.77, 95% CI 1.97–11.55; *p* < 0.001) when CMB count was entered as a continuous variable (OR 1.80 per microbleed, 95% CI 1.21–2.68; *p* = 0.004) and in the subgroup without prior stroke (adjusted OR 6.22, 95% CI 1.54–25.19; *p* = 0.010) (Supplementary Table S3).

**Table 2:** Crude and adjusted associations with MCI.

Variable	Crude OR (95% CI)	*P*	Adjusted OR (95% CI)	*P*
CMB burden (ordinal)	3.10 (1.75–5.50)	<0.001	4.53 (1.95–10.55)	<0.001
Age, per year	1.16 (1.11–1.22)	<0.001	1.16 (1.10–1.23)	<0.001
Male sex (vs. female)	0.69 (0.38–1.26)	0.229	0.80 (0.35–1.85)	0.604
Education, per year	0.84 (0.76–0.92)	<0.001	0.81 (0.71–0.91)	<0.001
SBP, per mmHg	0.99 (0.97–1.01)	0.491	0.99 (0.97–1.02)	0.562
Diabetes (yes vs. no)	0.78 (0.42–1.46)	0.438	0.77 (0.32–1.85)	0.559
Prior stroke (yes vs. no)	0.97 (0.53–1.78)	0.929	0.51 (0.22–1.19)	0.118

Odds ratios were estimated by logistic regression (*n* = 172; 83 MCI events). One participant with missing sex information was excluded from the adjusted analysis. The adjusted model included all listed variables simultaneously.

CMB burden was modeled as an ordinal variable (0, 1–4, ≥5 coded 0/1/2); continuous variables are expressed per unit increase; reference categories were female sex, absence of diabetes, and absence of prior stroke.

OR, odds ratio; CI, confidence interval; CMB, cerebral microbleed; SBP, systolic blood pressure; MCI, mild cognitive impairment.

For variables included in the primary multivariable model, tolerance values ranged from 0.794 to 0.942, and VIF values ranged from 1.062 to 1.259 (Supplementary Table S4). The joint Box–Tidwell test yielded *χ*^2^ = 7.291 with three degrees of freedom (*p* = 0.063). The Hosmer–Lemeshow test yielded *χ*^2^ = 6.564 with eight degrees of freedom (*p* = 0.584); the −2 log-likelihood was 148.247, and the Cox–Snell R^2^ and Nagelkerke *R*^2^ values were 0.407 and 0.543, respectively. Two observations had absolute standardized residuals greater than 3; the maximum leverage value was 0.177, and the maximum Cook’s distance was 0.105. No observation had a Cook’s distance greater than 1. After exclusion of the two observations, the adjusted OR for CMB burden was 5.99 (95% CI 2.38–15.06; *p* < 0.001) (Supplementary Table S5).

### Discrimination of MCI status

**Figure 5:**
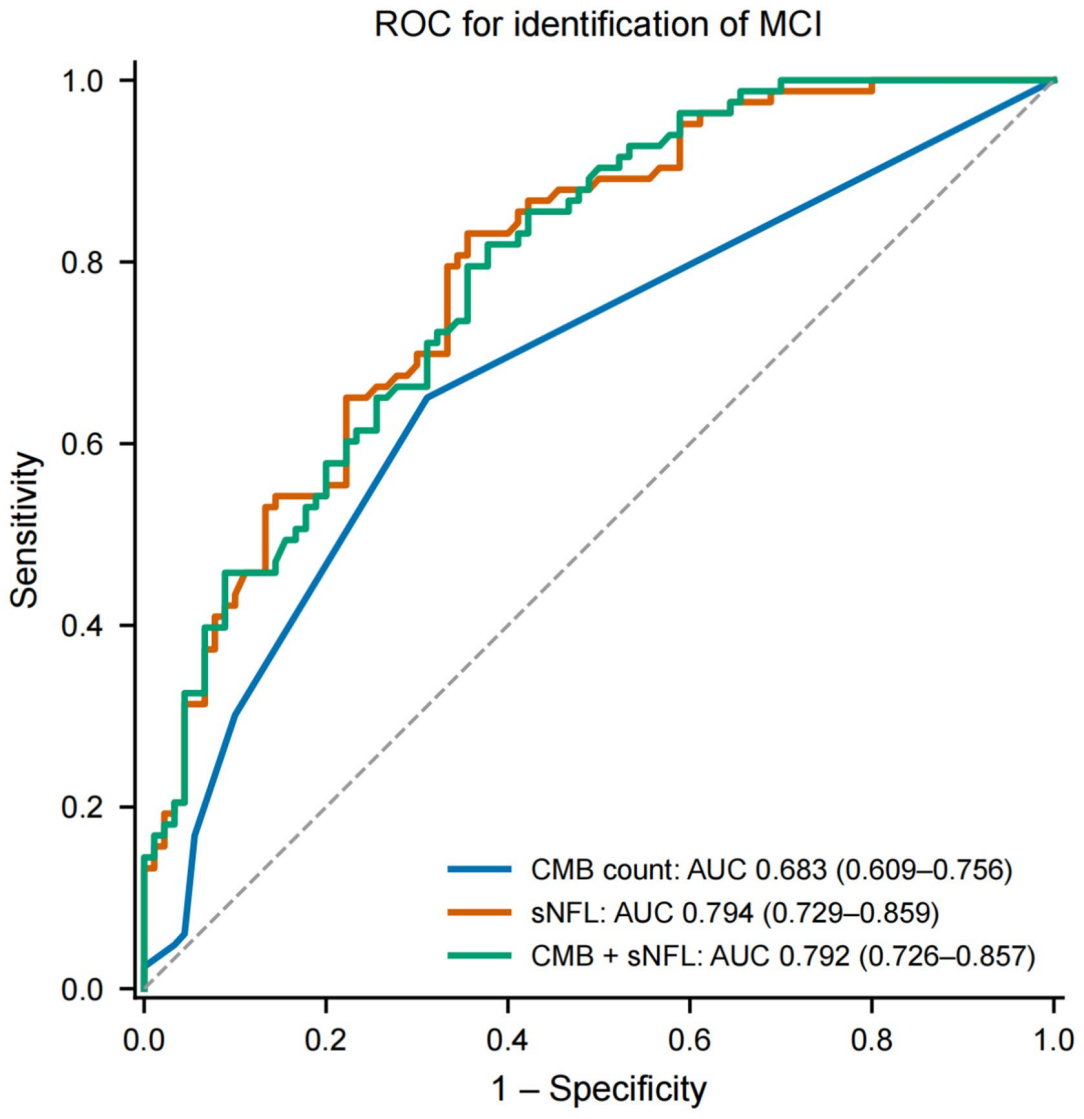
ROC curves of CMB count, sNFL, and the combined model for identifying MCI. Analyses included 173 participants. The combined model was derived from multivariable logistic regression incorporating CMB count and sNFL. ROC, receiver operating characteristic; AUC, area under the curve; CMB, cerebral microbleed; sNFL, serum neurofilament light chain; MCI, mild cognitive impairment.

The area under the ROC curve for identifying MCI was 0.683 (95% CI 0.609 to 0.756) for CMB count, 0.794 (95% CI 0.729 to 0.859) for sNFL, and 0.792 (95% CI 0.726 to 0.857) for the combined model (Figure 5). The combined model had a larger AUC than CMB count alone, with an AUC difference of 0.109 (95% CI 0.045–0.173; *Z* = 3.362; *p* < 0.001). The AUC difference between the combined model and sNFL alone was −0.002 (95% CI − 0.019 to 0.014; *Z* = −0.294; *p* = 0.768).

## Discussion

In this cross-sectional study of adults with primary hypertension, a higher cerebral microbleed burden was independently associated with MCI and with lower executive function, orientation, attention, and visuospatial function scores after adjustment and false discovery rate correction, whereas memory and language were not independently associated. CMB location was not associated with cognitive domain performance. The association remained when CMB count was modeled continuously. sNFL had a numerically higher AUC than CMB count, and adding CMB count to sNFL did not improve discrimination. Age and education were also independent correlates, whereas sex, systolic blood pressure, diabetes, and prior stroke were not independent correlates after adjustment.

The association between CMB burden and cognition was not uniform across domains. Unadjusted trend analyses showed lower scores across all six domains with increasing burden, whereas adjusted models retained associations with executive function, orientation, attention, and visuospatial function but not memory or language. In a clinic cohort of 563 participants, greater microbleed burden remained associated with poorer verbal fluency and with Stroop and maze performance after adjustment (26). In 688 patients with lacunar stroke, microbleeds were independently related to executive function and processing speed but not to memory, with adjusted odds of vascular cognitive impairment of 1.48 for their presence and 1.43 per additional lesion (27). Within hypertensive samples, five or more microbleeds have been linked to lower Montreal Cognitive Assessment scores and to slower information processing and motor speed (9), supporting an association between greater CMB burden and poorer cognition. Previous studies have frequently assessed processing speed, whereas the present MoCA-derived indices included orientation rather than a dedicated processing-speed measure. As one imaging manifestation of CSVD, CMBs may accompany injury to perforating arterioles and disruption of frontal–subcortical and other cognitive networks (1, 28). Iron released from microbleeds may contribute to local tissue damage, and quantitative susceptibility mapping has shown that patients with microbleeds carry a higher brain iron burden that relates independently to cognitive performance (29).

On the other hand, the absence of differences across microbleed locations contrasts with reports that emphasize topography. In a population-based study, microbleeds in deep or mixed regions showed the strongest links with decline in memory, processing speed, and executive function, and three or more lesions predicted steeper decline and a higher incidence of dementia (30). Mixed-location lesions in that cohort were most strongly related to memory and speed, whereas the location groups in the present study were small and uneven, with 11 lobar, 37 deep, 13 infratentorial, and 21 mixed cases. Deep and infratentorial lesions are attributed to hypertensive arteriopathy and strictly lobar lesions to cerebral amyloid angiopathy (6), but anatomical grouping may not fully represent the underlying vascular pathology in an individual participant. The use of MoCA-derived indices may also have reduced sensitivity to location-specific differences (23, 24). In this cohort, CMB burden showed a clearer association with cognition than anatomical location, although the null location findings do not exclude location-specific associations in larger samples with more balanced groups.

sNFL discriminated MCI with an area under the curve of 0.794, which is higher than the value of 0.646 reported for sNFL alone in a community sample screened for MCI and close to the value of 0.785 reported for plasma neurofilament light chain in post-stroke cognitive impairment (31, 32). The combined model did not exceed sNFL alone. The near-identical AUCs of sNFL alone and the combined model may reflect an overlap in the information captured by the two markers. sNFL concentrations have been associated with the overall small vessel disease load, including microbleeds, lacunes, and white matter hyperintensities, with reported odds ratios of 1.29 for microbleeds and 1.67 for moderate to severe white matter hyperintensities (33), and higher levels accompany greater white matter damage and atrophy in patients with small vessel disease (34). Because NfL reflects neuroaxonal injury associated with several brain pathologies, it may capture tissue injury related to CMBs and other lesions, leaving limited additional information from CMB count once sNFL is included. sNFL also carries prognostic weight in small vessel disease, where baseline levels predict conversion to dementia with a hazard ratio of 1.68 and relate to long-term mortality (35, 36), and it has been proposed as a blood-based marker of disease severity (36). Although blood sampling is more accessible than MRI, sNFL lacks anatomical and etiological specificity and should not be considered a substitute for MRI, which depicts CMB location and other CSVD markers. The AUC observed in this study and the absence of an externally validated threshold do not support immediate use of sNFL as a stand-alone clinical test.

Blood pressure, diabetes, and prior stroke were associated with microbleed burden in the unadjusted comparison but were not independently associated with MCI after adjusting for microbleed burden and other covariates. These findings indicate that their associations with cognitive status were not independent of the other variables in the model and do not identify a mediating pathway. Associations among vascular exposures, CSVD markers, and cognition have been reported in other cohorts (37), but mediation was not evaluated in the present analysis. The adjusted estimate for prior stroke was imprecise and not statistically significant and should not be interpreted as protective. The low variance inflation factors do not support multicollinearity as an explanation; sampling variability, residual confounding, and the high prevalence of prior stroke may have contributed to this estimate.

The association between microbleed burden and MCI persisted after adjusting for white matter hyperintensity grade, lacune count, and perivascular space grade, after modeling microbleeds as a continuous variable, and after restricting the analysis to patients without prior stroke. It also remained after exclusion of the two observations with absolute standardized residuals greater than 3. These analyses support the stability of the association across alternative covariate adjustment, exposure coding, subgroup restriction, and influence diagnostics. Persistence after adjustment for the measured CSVD markers suggests that the association was not fully accounted for by Fazekas grade, lacunes, and perivascular spaces (27). The overlap between microbleeds and the wider small vessel disease spectrum nonetheless remains substantial. Only seven participants had at least five CMBs, which limited precision at the highest burden level and restricted assessment of the dose–response pattern. The continuous-count analysis supported the primary association but did not remove the uncertainty associated with the small high-burden group.

Quantifying microbleed burden on susceptibility-weighted imaging or measuring sNFL may provide additional information regarding concurrent cognitive vulnerability in hypertensive adults, although their potential role in clinical screening requires further validation. Intensive blood pressure lowering reduced the occurrence of MCI and the composite of MCI or dementia compared with standard control in a randomized clinical trial (3), along with careful vascular risk management in patients with hypertension. The present findings do not define screening thresholds or support treatment decisions based on CMB burden or sNFL. Longitudinal validation is required to determine whether either marker precedes cognitive decline or improves clinical decision-making.

This study has several strengths, including a hypertension-specific sample, characterization of cognition at the level of individual domains, the pairing of an imaging marker with a blood marker, standardized microbleed rating by two blinded readers, and consistent results across sensitivity analyses. Several limitations qualify the findings. The cross-sectional design cannot establish temporal or causal relationships between microbleed burden and cognition. It also precludes the assessment of predictive performance. The sample came from a single center, spanned a wide age range, and included a high proportion of patients with prior stroke, and hospital-based recruitment may limit generalizability to community populations. These features also leave potential for residual confounding. Cognitive domains were derived from the Montreal Cognitive Assessment rather than a dedicated battery. Although the validity of MoCA-derived index scores has been evaluated against domain scores from neuropsychological batteries, these indices do not replace domain-specific testing. The MCI definition relied largely on an education-adjusted MoCA threshold together with preserved activities of daily living and absence of clinical dementia, which may have introduced classification error. The small and uneven location groups and the seven-participant high-burden group reduced precision for subgroup analyses. sNFL was measured with an enzyme-linked immunosorbent assay, which has lower analytical sensitivity than ultrasensitive platforms such as single-molecule array technology (38). The ROC models were not externally validated. Future studies should include longitudinal follow-up, larger multicenter cohorts with more balanced CMB groups, domain-specific neuropsychological testing, ultrasensitive sNFL assays, and external validation to determine whether CMB burden and sNFL are associated with subsequent cognitive decline.

## Conclusion

In this cohort of adults with primary hypertension, greater CMB burden was independently associated with MCI and with lower executive function, orientation, attention, and visuospatial function scores, whereas CMB location was not associated with cognitive domain performance. sNFL had a numerically higher AUC for identifying MCI than CMB count, while adding CMB count to sNFL did not improve discrimination. These cross-sectional findings demonstrate associations and do not establish predictive or clinical utility. Prospective longitudinal studies are needed to determine whether CMB burden and sNFL predict cognitive decline over time.

## Data Availability

The original contributions presented in the study are included in the article/Supplementary material, further inquiries can be directed to the corresponding author.
